# A phase 1b study on the safety of Wharton’s jelly mesenchymal stromal cells in the treatment of acute graft-versus-host disease

**DOI:** 10.1016/j.jcyt.2025.102012

**Published:** 2025-11-26

**Authors:** William Wesson, Jianzheng Wu, Milind Phadnis, Scott Weir, Tyce Bruns, Ramesh Balusu, Haitham Abdelhakim, Rupal Soder, Sunil Abhyankar, Joseph P. McGuirk

**Affiliations:** 1 University of Kansas Medical Center, Kansas City, Kansas, USA; 2 University of Kansas Department of Statistics, Kansas City, Kansas, USA; 3 University of Kansas Institute for Advancing Medical Innovation, Kansas City, Kansas, USA; 4 Midwest Stem Cell Therapy Center, University of Kansas Medical Center, Kansas City, Kansas, USA

**Keywords:** graft-versus-host disease, steroid-refractory, Wharton’s jelly, Mesenchymal stromal cells, stem cell therapy

## Abstract

**Background aims:**

Steroid-refractory (SR) and high-risk (HR) acute graft-versus-host disease (aGVHD) is a major complication of allogeneic hematopoietic stem cell transplantation. Mesenchymal stromal cells (MSCs) possess immunomodulatory potential, and Wharton’s jelly MSCs, a rapidly expanding subset of cells that exhibit potent *in vitro* suppression of T-cell proliferation, may benefit patients in the setting of SR/HR aGVHD. The objective of this study was to evaluate the safety and preliminary efficacy signals of multiple-dose MSCTC-0010, an umbilical cord–derived Wharton’s jelly MSC product, in patients with SR or HR aGVHD.

**Methods:**

This was a prospective, single-center, open-label phase 1b trial (NCT03158896). Adults (18–76 years old) with *de novo* HR or SR aGVHD unresponsive to ≥1.6 mg kg^−1^ day^−1^ methylprednisolone were enrolled. Three sequential cohorts received 2 × 10^6^ (low dose [n = 5]), 10 × 10^6^ (high dose [n = 5]) or 10 × 10^6^ cells kg^−1^ on day 0, day 7, day 14 and day 21 (extended high dose [n = 14]) intravenously. The primary endpoint was the absence of treatment-related adverse events (TRAEs) (Common Terminology Criteria for Adverse Events grade ≥3 persisting >24 h) by day 42. Secondary endpoints included overall response (OR) and complete response (CR) at day 28 and day 90, need for additional immunosuppression and ectopic tissue formation as detected by computed tomography (CT) on day 90. Analyses were descriptive in intention-to-treat (all treated) and at-risk (alive/on-study) populations.

**Results:**

Twenty-four patients received an infusion; median age was 59 years and 66% were male. No TRAEs were observed. One patient experienced transient infusion-related dyspnea. No ectopic tissue formation was detected on CT imaging at day 90.

By day 28, 71% of patients demonstrated clinical improvement: 33% achieved CR and 38% achieved a partial response (PR). By day 90, 54% of patients showed improvement (CR 42%, PR 13%). Median time to additional systemic therapy (ruxolitinib ± extracorporeal photopheresis) was 15 days; 75% ultimately required escalation. Median overall survival was 7.0 months for all patients and 30 months for those completing all planned doses.

**Conclusions:**

MSCTC-0010 demonstrated an excellent safety profile with no grade ≥3 TRAEs and no ectopic tissue formation. Promising early efficacy—including 71% day 28 OR and 42% day 90 CR in a population with historically poor outcomes—supports further investigation. A randomized multicenter phase 2 trial combining or sequencing MSCTC-0010 with ruxolitinib is warranted to clarify its therapeutic contribution in HR/SR aGVHD.

## Introduction

Allogeneic hematopoietic stem cell transplantation (HSCT) has become an increasingly used treatment modality, with over 90 000 transplants performed annually across the globe [1]. Although allogeneic HSCT represents a curative option for many hematologic malignancies, it is associated with substantial early and late treatment-related morbidity and mortality. Post-transplant complications, including infection, organ toxicity and graft-versus-host disease (GVHD), are the predominant drivers of non-relapse mortality [2].

Acute GVHD (aGVHD), which occurs most frequently within the first several months post-transplant, is mediated by donor-derived T-cell inflammation in the skin, liver and gastrointestinal tract due to disparities in major and minor histocompatibility antigens between donor and recipient tissues [3]. Despite advances in prophylactic strategies, aGVHD remains a common and serious complication of allogeneic HSCT.

Systemic corticosteroids remain the first-line therapy for aGVHD, with limited exceptions, such as for isolated low-grade cutaneous manifestations, which can be treated with topical corticosteroids [4]. However, patients who fail to respond to systemic steroid treatment face a poor prognosis. A pivotal 2020 clinical trial demonstrated that ruxolitinib significantly improved overall response (OR) rates at day 28 compared with other commonly used second-line agents, leading to its approval and adoption as second-line therapy for steroid-refractory (SR) aGVHD [5]. Nonetheless, by day 56, approximately 60% of patients required additional therapy or had died, underscoring the significant morbidity and mortality inherent in this disease population. Additionally, ruxolitinib was shown to have significant toxicity, with all patients developing treatment-emergent adverse events and 97% of patients having grade 3 or higher treatment-emergent adverse events (mostly cytopenias and infections), underscoring the toxicity inherent to immune suppression in this setting [6]. Although clinical responses to steroids in aGVHD patients have been a strong predictor of outcomes including mortality, the clinically validated Minnesota criteria have been developed to identify high-risk (HR) aGVHD based on clinical criteria at diagnosis [7]. HR aGVHD patients who are identified as steroid-naive by the Minnesota criteria have worse outcomes, including worse mortality, comparable to SR aGVHD patients [8]. Several aGVHD clinical trials have included patients identified by this algorithm, including the BMT CTN1501 trial [9]. We opted to include patients with HR aGVHD in this clinical trial, as these patients have high odds of developing SR aGVHD and their outcomes are comparable to those of SR aGVHD patients.

Efficacious second- and third-line agents for SR aGVHD remain sparse. On December 18, 2024, the US Food and Drug Administration (FDA) approved the first mesenchymal stromal cell (MSC) therapy, remestemcel-L-rknd, for treatment of SR aGVHD in children 2 months and older [10]. Remestemcel-L-rknd is an infusion of HLA–non-matched bone marrow–derived MSCs. MSCs can be employed in various inflammatory conditions, including GVHD, because of their immunosuppressive properties. MSCs inhibit T-cell activation and proliferation through immunomodulatory mechanisms such as cytokine production, T-cell inhibitory extracellular vesicles and contact inhibition [11].

Adult-derived MSCs may have reduced expansion potential and immunosuppressive properties compared with fetal or neonatal MSCs [12]. Discarded post-natal tissues may offer an additional advantage for easier MSC collection compared with bone marrow–derived MSCs. Wharton’s jelly, a primitive connective tissue rich in hyaluronic acid, supports the umbilical vessels. After birth, it contains an easily isolated MSC population. Wharton’s jelly MSCs (WJMSCs) grow faster and produce more cells *in vitro* than bone marrow–derived MSCs [13]. WJMSCs also have immunosuppressive properties comparable to adult-derived MSCs from bone marrow and adipose tissue.

In this phase 1b clinical trial, we expand on the safety outcomes and describe early signals of efficacy for MSCTC-0010, a WJMSC cell product, for the treatment of SR and HR aGVHD in accordance with Strengthening the Reporting of Observational Studies in Epidemiology guidelines [14]. We previously reported the results from the first 10 patients in the phase 1a clinical trial [15].

## Methods

### Patients and trial design

This single-center, prospective, open-label phase 1b cohort trial (ClinicalTrials.gov no. NCT03158896, investigational new drug no. 017672) was designed to evaluate the safety and efficacy of multiple doses of umbilical cord–derived *ex vivo* cultured and expanded WJMSCs (MSCTC-0010) when administered to participants with *de novo* HR or SR aGVHD post-allogeneic stem cell transplant. The study was approved by the institutional review board and was conducted in accordance with the principles of the Declaration of Helsinki and the International Conference on Harmonization Good Clinical Practice guidelines. Written informed consent was obtained from the patients prior to enrollment in the study and treatment and was subject to institutional review board review.

Adult patients aged 18–76 treated at the University of Kansas Cancer Center were eligible for participation as SR if they failed to respond to systemic steroid treatment as first-line treatment for aGVHD, defined as progression within 3 days of initial treatment or no improvement within 7 days of consecutive treatment with 1.6 mg/kg/d of methylprednisolone or equivalent. High risk was defined by Minnesota criteria (see supplementary Figure 1) [7]. Patients were excluded if they had received any other investigational agent used to treat aGVHD within 30 days prior to enrollment.

### Investigational product

MSCTC-0010 suspension for infusion is a suspension of WJMSCs from umbilical cord tissue manufactured by the Midwest Stem Cell Therapy Center under investigational new drug number 017672. Expectant mothers at the University of Kansas Medical Center planning to undergo elective cesarean section provided written informed consent for the donation of umbilical cords. The umbilical cords were accepted from healthy full-term women. In order to qualify as cord donors, mothers were tested for human immunodeficiency virus types 1 and 2; hepatitis A, B and C; *Treponema pallidum; Chlamydia trachomatis; Neisseria gonorrhoeae*; and human T-cell lymphotropic virus 1 and 2. From these donated cords, WJMSCs were isolated, cultured and expanded under current Good Manufacturing Practice/Good Tissue Practice standards as previously reported [15,16].

Cells were characterized for MSC-expressed antigens by flow cytometry using the BD Stemflow human MSC analysis kit (BD Biosciences, San Diego, CA, USA). Flow cytometry revealed that ≥95% of cells expressed the markers characteristic of MSCs (CD105, CD73, CD90 and CD44), whereas the expression of hematopoietic, macrophage and B-cell markers (CD45, CD34, CD11b, CD19 and HLA-DR) was 2% or less. We examined the ability of MSCTC-0010 to suppress proliferation of activated human peripheral blood mononuclear cells (PBMCs). PBMCs were labeled with 5 *μ*M carboxyfluorescein succinimidyl ester (Thermo Fisher Scientific, Waltham, MA, USA), stimulated with 1 *μ*g/mL phytohemagglutinin (Sigma-Aldrich, St Louis, MO, USA) and co-cultured with MSCTC-0010 at a ratio of 1:10 for 3 days. The intensity of carboxyfluorescein succinimidyl ester staining of phytohemagglutinin-induced proliferating PBMCs was evaluated by flow cytometry (BD LSR II flow cytometer; BD Biosciences). Data were analyzed using BD FACSDiva software (BD Biosciences). MSCTC-0010 cells suppressed mitogen-stimulated PBMC activation and proliferation. MSCTC-0010 was determined to be sterile (USP<71> sterility test) and endotoxin-free using a quality control test kit (*Limulus* amebocyte lysate method, Endosafe system; Charles River Laboratories, Wilmington, MA, USA). MSCTC-0010 was negative for mycoplasma (MycoAlert mycoplasma detection kit; Lonza, Basel, Switzerland) and had no chromosomal abnormalities (cytogenetic analysis; University of Kansas Medical Center). MSCs were harvested after passage five. The final product was cryopreserved in PlasmaLyte A (Baxter, Deerfield, IL, USA) supplemented with dimethyl sulfoxide and human serum albumin and thawed immediately prior to administration, with a cell viability ≥80% as the final release qualification.

The rationale for the dosing of the initial 10 patients was based on prior studies demonstrating responses at doses of 2 × 10^6^ MSCs/kg [17]. Subsequent patients were treated with repeated doses based on studies demonstrating sustained responses with fewer flare-ups of aGVHD with this strategy [18–22]. The first cohort of five patients therefore received an intravenous dose of 2 × 10^6^ viable MSCs/kg on day 0 and day 7 (low-dose cohort). The second cohort of five patients received a dose of 10 × 10^6^ viable MSCs/kg on day 0 and day 7 (high-dose cohort). The rationale for the higher dose was based on both pre-clinical and clinical investigations demonstrating safety at higher doses of 8 × 10^6^ MSCs/kg [17]. The subsequent 10 patients were scheduled to receive a dose of 10 × 10^6^ viable MSCs/kg on day 0, day 7, day 14 and day 21 (extended high-dose cohort) based on aGVHD biomarker analysis from the first 10 patients showing improvement in the biomarker score with the higher dose of MSCTC-0010 [15]. Because of progressive disease, four patients in the initial extended high-dose cohort did not receive all scheduled doses; therefore, an additional four patients were recruited into the extended high-dose cohort for a total of 24 MSCTC-0010–infused patients. This allowed us to have a total of 20 patients who received all planned doses of MSCTC-0010.

The first dose was given in the first 10 patients within 5 days of diagnosis of either HR or SR aGVHD; all remaining patients were SR. Prior to infusion, patients were given hydrocortisone 25–50 mg intravenously and diphenhydramine 25–50 mg orally or intravenously to prevent infusion reactions. During the trial, patients maintained their baseline established systemic steroid therapy based on institutional algorithms and their GVHD prophylactic agent. In the event of GVHD progression in the 7 days after initial infusion, additional medications for the treatment of aGVHD were allowed. Each patient received an infusion of cells derived from distinct cords in the manufacture of MSCTC-0010; thus, 24 umbilical cords were used in the manufacture of 24 full doses.

### Assessment of safety and efficacy

GVHD assessments were performed by delegated investigators weekly from enrollment through day 42 and on day 90. The severity of aGVHD for each patient was evaluated using the Consensus Conference on Acute GVHD Grading scale [23]. All untoward medical occurrences after the initial treatment were considered adverse events. Adverse events were graded based on the Common Terminology Criteria for Adverse Events (version 5.0) [24].

### Study endpoints

The primary endpoint was the proportion of participants who reached day 42 after infusion of MSCTC-0010 without a treatment-related adverse event, defined as a grade 3 or greater adverse event that was at least probably related to the investigational product and that did not return to baseline within 24 h of the start of the event. Secondary endpoints were the proportions of participants who achieved a complete response (CR) to aGVHD by day 28 and day 90, experienced improvement in aGVHD by day 28 and day 90 in one or more involved organs, required the addition of escalated systemic immunosuppressive therapy within 90 days of the first MSCTC-0010 dose and demonstrated the formation of ectopic tissue foci at day 90, as evaluated by computed tomography scans of the chest, abdomen and pelvis.

### Statistical analysis

Data cutoff for analysis was April 20, 2025. Endpoints were analyzed and reported on using descriptive statistics and frequency tables in both intention-to-treat (all) and at-risk populations, defined as patients who had not died or come off the study. Survival was described using Kaplan–Meier survival curves. The aGVHD response was measured by improvement in at least one affected organ system (partial response [PR]), complete resolution of disease in all organ systems (CR), no change in disease involvement (no response) or progressive disease after initial response, as evaluated by clinical investigators. OR was defined as either CR or PR.

## Results

An initial 20 patients were enrolled in the study and treated between August 2018 and January 2024, with results from the first 10 patients having been previously reported [15]. Several patients (n = 4) in the subsequent cohort had progressive disease or serious infections and came off the study prior to receiving their full allotted dose schedule of MSCTC-0010; therefore, the cohort was expanded until 20 patients completed their full allotted treatment in this expanded study. To attain 20 patients who completed all allocated doses, 24 patients received MSCTC-0010 and were included for analysis. The additional four patients past the originally planned 20 were treated in the extended high-dose cohort. The median age of patients was 59 years (range, 35–76 years). Sixteen of the patients (66%) were male (Table 1). At enrollment, one patient was high risk, 13 were SR and 10 were both high risk and SR. The one exclusively HR patient was initiated on steroids concurrently with the infusion of MSCTC-0010.

### Safety outcomes

No patients experienced a treatment-related adverse event during the follow-up period. One patient experienced infusion-related shortness of breath, attributed to infusion of MSCTC-0010, but the event resolved quickly and the patient returned to baseline with no sequelae. At day 90, patients were evaluated for the formation of ectopic tissue foci using computed tomography scans of the chest, abdomen and pelvis. Of the 15 evaluable patients, none had any ectopic tissue detected.

### Efficacy outcomes

By day 28 following the first infusion, 19 patients were evaluable for outcomes. Seventeen patients (71% of all, 89% of at-risk) had OR in at least one organ system involved at baseline (see supplementary Table 1). Of these 17 patients, eight (33% of all, 42% of at-risk) had CR and nine (38% of all, 47% of at-risk) had PR. Two patients (8% of all, 11% of at-risk) had no response and five (21% of all) had progressed and come off the trial or died by day 28. Responses by dose cohort are shown in supplementary Figure 2.

**Fig. 1.**
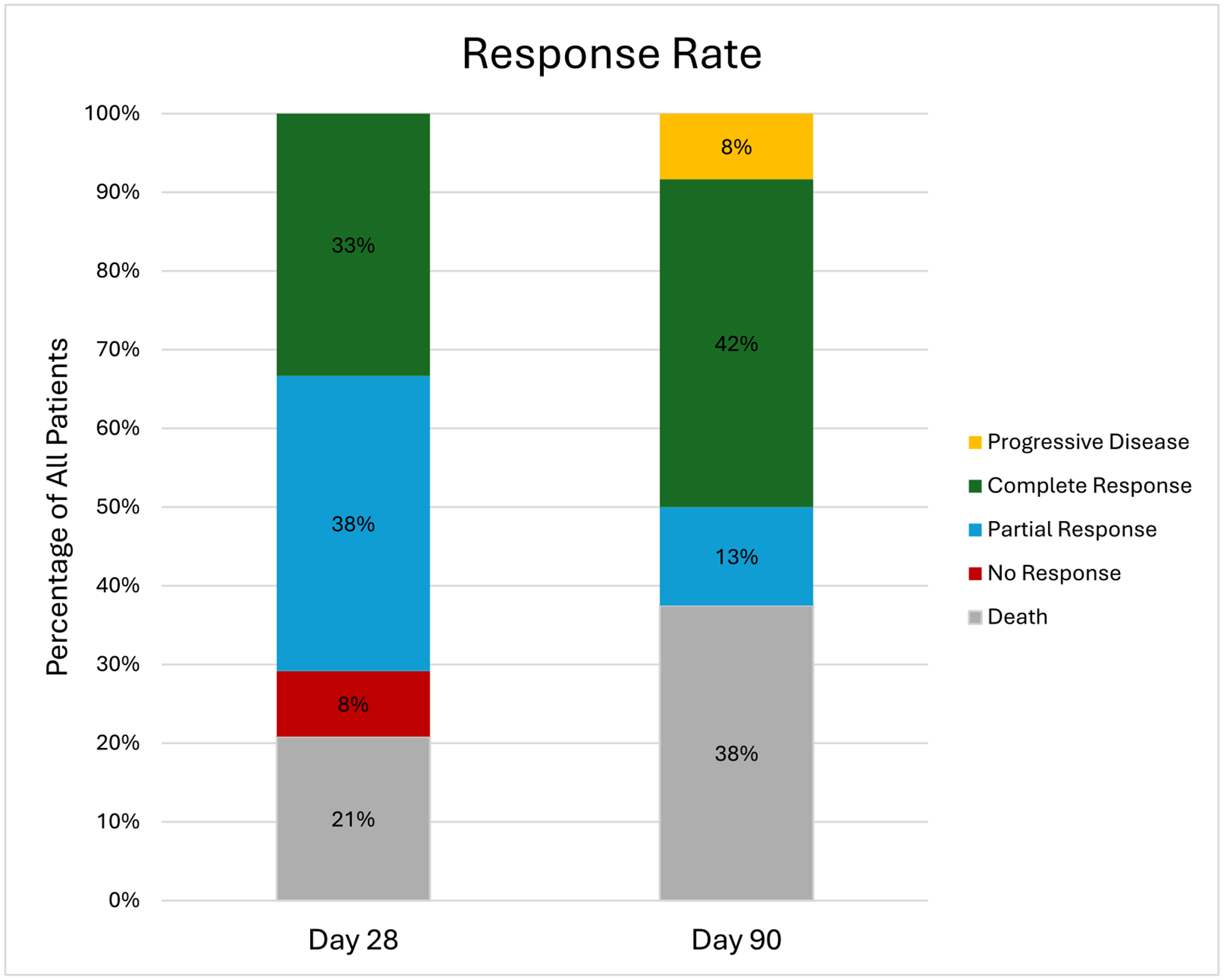
Response to treatment at day 28 and day 90.

By day 90 following the first infusion, 15 patients were evaluable for outcomes. Thirteen patients (54% of all, 87% of at-risk) had OR in at least one organ system involved at baseline. Of these 13 patients, 10 (42% of all, 67% of at-risk) had CR and three (13% of all, 20% of at-risk) had PR (Figure 1). Eleven patients (46% of all) had progressed to a non-response and come off the trial or died by day 90. Of the nine patients who had achieved PR by day 28, six (67%) achieved CR at day 90, one (11%) had progressive aGVHD and was still living at day 90 and two (22%) had progressed and withdrawn from the study by day 90. Three patients (13% of all, 20% of at-risk) had a decrease in response at day 90 from prior CR at day 28 to PR at day 90 but were still improved from baseline.

Within the first 90 days following first infusion of MSCTC-0010, 18 patients (75% of all) had additional immunosuppressive therapy added as adjuvant therapy for aGVHD, with all of these patients receiving ruxolitinib in addition to six patients (25% of all) receiving extracorporeal photopheresis. The median time to initiation of additional immunosuppressive therapy was 15 days from MSCTC-0010 infusion.

### Long-term outcomes

At day 180 following first infusion, 12 patients (50% of all) were evaluable and 12 patients (50% of all) were deceased. Of the 12 patients who had reached day 180, eight had developed chronic GVHD. Six patients (25% of all, 50% of at-risk) developed extensive chronic GVHD and two (8% of all, 17% of at-risk) developed limited chronic GVHD. Of the eight patients who attained CR on day 28, seven (88%) survived to day 180. Of the nine patients who attained PR on day 28, five (56%) survived to day 180. One patient who had no response to therapy withdrew from the study but nevertheless survived to day 180.

**Fig. 2.**
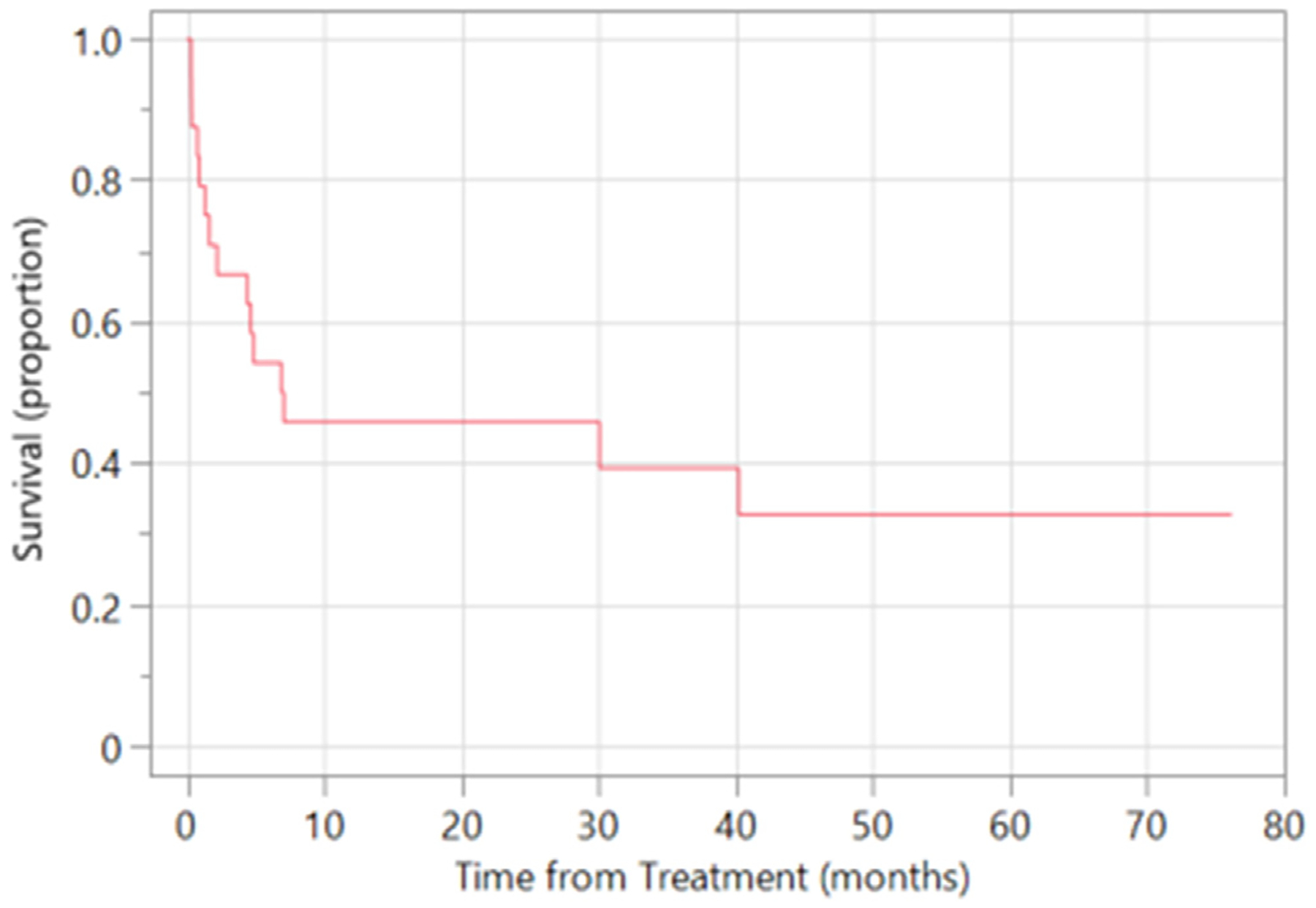
Patient overall survival.

The median follow-up for all trial patients was 7 months (range, 8 days to 76 months). For patients who received all allocated doses, median follow-up was 14 months (range, 26 days to 76 months). A total of 15 patients (63%) died at some point after infusion, with a median survival of 7.0 months (interquartile range, 1.5 months to not reached) (Figure 2). The median survival of patients who received all allocated doses was 30 months (interquartile range, 4.5 months to not reached). Of the 15 patients who died during follow-up by the data cutoff, six died of complications from aGVHD, four died of infection, three died of concurrent aGVHD and infection and two died of complications from primary disease progression. Three patients (13%) had a relapse of their primary malignancy in the follow-up period. The infections resulting in death included bacterial, fungal and viral pneumonia as well as bacterial and viral sepsis.

## Discussion

In this single-center phase 1b trial of MSCTC-0010, none of the 24 treated patients were found to have experienced a treatment-related adverse event, suggesting a favorable safety profile. Additionally, by day 28, 71% (17 of 24) of all patients had OR to treatment and 33% (eight of 24) had CR with no residual aGVHD symptoms. By day 90, just under half of all patients had achieved CR. In combination, these results could be viewed as early signals of efficacy.

As a novel therapy for the treatment of HR/SR aGVHD, MSCTC-0010 has promise as a secondary or tertiary agent. MSCs express several unique immunomodulatory properties *in vitro* and have been used in multiple inflammatory conditions in humans, including aGVHD [11]. Importantly, MSCs have been shown to suppress T-cell proliferation, shift pro-inflammatory T helper 1 cells to anti-inflammatory T helper 2 cells and promote differentiation of monocyte/macrophage and dendritic cells into anti-inflammatory phenotypes [25–27]. Subsequent studies have reported that adult-derived MSCs and WJMSCs have similar immunosuppressive and anti-inflammatory effects [28–31]. After intravenous administration, WJMSCs do not home to injured tissue as they are trapped in the pulmonary capillary bed [32]. The secretion of bioactive exosomes may, in part, explain the systemic responses post-intravenous infusion of WJMSCs. We have previously reported an increase in exosomes carrying programmed death ligand 1 post-WJMSC infusion in patients with aGVHD [33]. Further work in biomarker, cytokine and other translational science with WJMSCs is under way and forthcoming in a future article. One unique hypothetical safety issue related to MSC therapy is the potential for developing ectopic tissue given that MSCs are third-party adherent cells and have the hypothetical potential to travel to the lungs and other areas of the body, evade the immune system and grow into ectopic tissue [34]. However, previous clinical trials have failed to detect any ectopic tissue, and our study further supports these findings [17,35,36].

Patients who fall into the classification of HR/SR aGVHD are traditionally at an immensely increased risk of mortality. A study by Ardizzoia *et al*. [37] demonstrated that 2-year overall survival in Minnesota HR patients was substantially reduced from that in standard-risk patients (30.7% compared with 57%), resulting in mortality of nearly 70% just 2 years post-allogeneic stem cell transplant regardless of systemic corticosteroid use. There is a similarly dismal prognosis for SR aGVHD, with mortality at 2 years between 70% and 83% [38–40]. For patients facing such poor outcomes even after the otherwise successful treatment of a primary malignancy, earnest research is required to address the gap in efficacious therapeutic options.

In 2021, ruxolitinib, a Janus kinase 1/2 inhibitor, was FDA-approved as a second-line agent for aGVHD after failure of systemic corticosteroids based on the REACH1 trial [41]. In that pivotal trial, 71 SR aGVHD patients were treated with ruxolitinib [6]. At day 28 following ruxolitinib treatment, OR was nearly 55%, with just over 26% of patients showing CR. Patients had treatment-emergent adverse events similar to previous trials involving ruxolitinib [42–44]. Importantly, treatment-related adverse events, including cytopenias, were tied for the leading cause of therapy discontinuation or dose adjustment with physician decision. Despite positive results for SR patients that resulted in FDA approval of ruxolitinib, in the follow-up REACH2 trial, it was demonstrated that mortality continued to be high (approximately 50%) regardless of ruxolitinib use, with a majority of deaths attributed to aGVHD followed by underlying disease progression [5]. In our trial, almost all of the initial 10 enrolled patients had not received ruxolitinib prior to infusion with MSCTC-0010, with the exception of two patients who received it in the course of treatment for myelofibrosis (another indication for ruxolitinib), as enrollment for these patients occurred prior to FDA approval of ruxolitinib for aGVHD. The subsequent cohort enrollment occurred after FDA approval, and as such the second 14 patients had nearly ubiquitously received ruxolitinib prior to MSCTC-0010, with the exception of three patients who were unable to receive it because of logistical issues.

With such poor outcomes as a baseline, there are numerous ongoing trials with novel therapeutic options for the treatment of HR/SR aGVHD aiming to fill the therapeutic gap by addressing unique mechanisms in the pathogenesis of aGVHD, although outcomes can be difficult to compare side by side after the recent approval of ruxolitinib as a second-line agent. Recombinant interleukins and several monoclonal antibodies are among the agents in early-phase clinical trials that are demonstrating promise as possible third-line agents for aGVHD [45–47]. Additionally, there are WJMSC derivative products (e.g., exosomes) that share similar immunomodulatory properties to WJMSCs that are under early investigation [33,48].

Although our study is novel, MSCs are hardly new in the therapeutic space of hematopoietic malignancies. Studies as early as 2004 demonstrated the efficacy of MSCs for severe GVHD [49]. Studies in subsequent years have demonstrated both efficacy in the treatment of GVHD and a very impressive safety profile, with meta-analyses consistently affirming no evidence of infusion-related toxicities, increased risk of underlying disease relapse or malignant transformation [35,50–53]. Compared with other pharmaceuticals in this space, MSCs demonstrate therapeutic potential with a greatly reduced adverse event profile. This beneficial profile, along with study data demonstrating OR of 70% at day 28 following infusion, led to FDA approval of remestemcel-L-rknd for pediatric patients with SR aGVHD after HSCT in December 2024 [10]. Dosing of remestemcel-L-rknd at 2 × 10^6^ MSCs/kg twice weekly for 4 consecutive weeks for a total of at least eight infusions, although spread over a longer period of time, represents a lower overall dose compared with our study. Following FDA approval in pediatric populations, this may inform dosing in future WJMSC trials.

Despite the inherent risks of aGVHD, there is a careful balance to be struck between control of aGVHD symptoms and complete immunosuppression and subsequent loss of beneficial graft-versus-tumor effect [54]. This graft-versus-tumor reactivity is one of the main advantages of HSCT, and over-suppression of the immune system in an effort to control GVHD can in turn lead to underlying disease relapse; therefore, the more refined and discriminating our therapies can be in targeting allo-reactive T cells, the better our long-term outcomes in both survival and primary disease relapse will be [55].

**Fig. 3.**
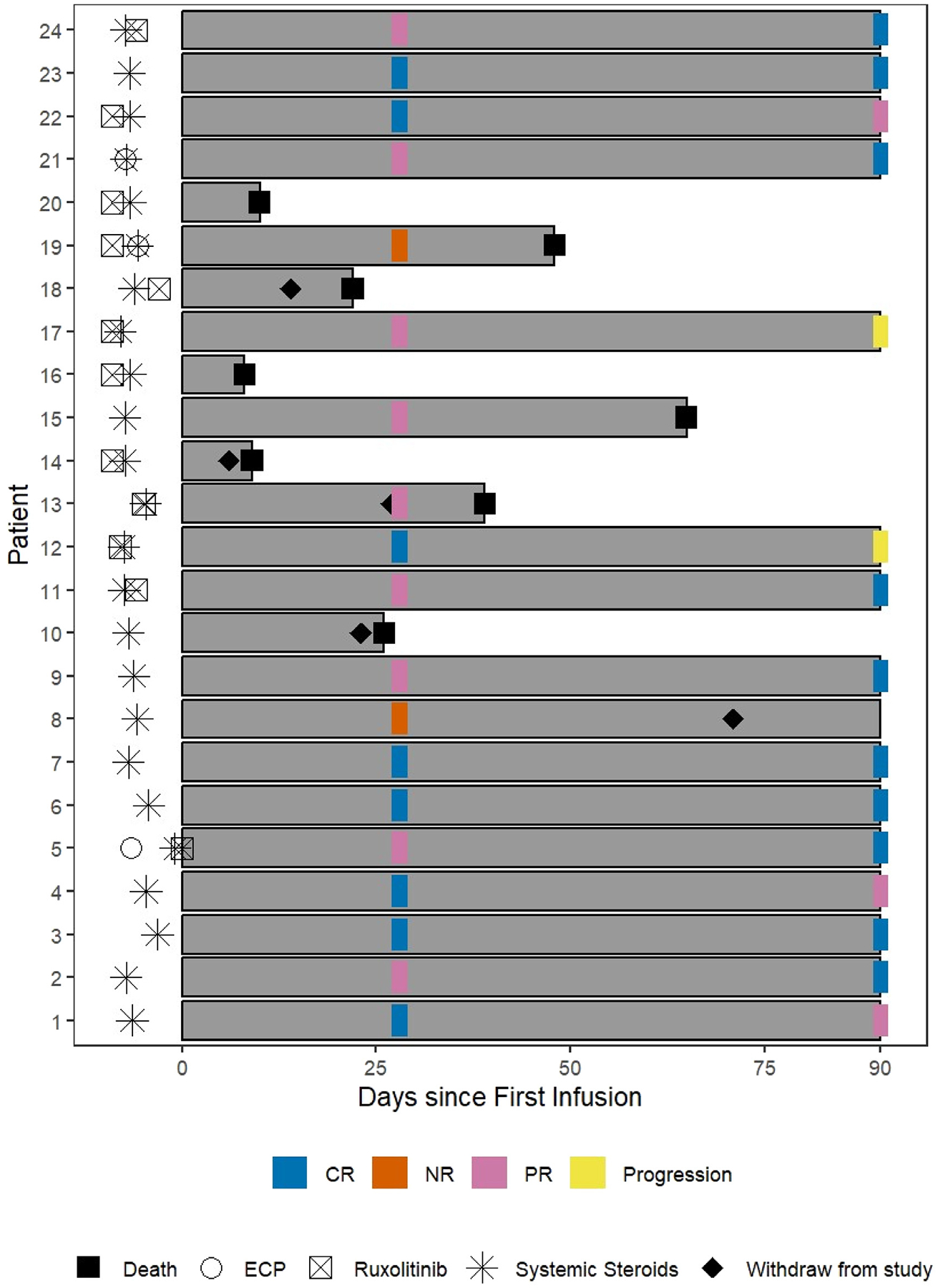
Swimmer plot of aGVHD therapies and response through day 90. ECP, extracorporeal photopheresis; NR, no response.

A limitation of all analyses in this study is the limited patient numbers. An additional limitation of our study is that survival and outcome data could be influenced by the addition of third-line (and beyond) agents, seen in 18 trial patients (75%). These additional agents were added early, with a median time of 15 days to the addition of any next agent after MSCTC-0010 infusion, owing to the aggressive nature of HR/SR aGVHD, and could be confounders of the assessments of efficacy in this trial (Figure 3). Additionally, this non-randomized trial did not allow for comparison with upfront ruxolitinib-treated patients, as FDA approval of that agent was announced partway through this study. It is conceivable that patients who have failed ruxolitinib might have even poorer outcomes compared with those with purely SR aGVHD. Efficacy outcomes are difficult to assess with the addition of ruxolitinib as an FDA-approved second-line agent partway through this study, as the approval necessitated a change in prescribing practice during the conduct of this clinical trial. Finally, efficacy outcomes might be different between dose cohorts, but limited patient numbers in this phase 1b trial prevent a meaningful analysis between these cohorts.

Based on the results of our study, expanding to a phase 2 multicenter study with ruxolitinib in the second line of therapy with or without WJMSCs would be reasonable and offer an excellent new therapeutic strategy in patients with HR or SR aGVHD, who traditionally have severe morbidity and mortality. It has been demonstrated that MSCs are effective in an active inflammatory environment, and therefore it can be intuited that the activation and efficacy of MSCs might be limited when given concurrently with ruxolitinib [56,57]. However, we propose that a significant proportion of patients have aGVHD that progresses through ruxolitinib therapy, indicating that a significant proportion of patients have active inflammation despite the downregulation of inflammatory pathways by Janus kinase inhibitors. New manufacturing modalities have been under development at the Midwest Stem Cell Therapy Center to improve the efficacy of WJMSCs for each umbilical cord, aiming to improve MSCTC-0010 production while preserving the quality of the final product. To that end, additional quality control tests are being validated in ongoing studies. Future studies to expand on the use of combinations of agents are warranted. Just as we approach induction chemotherapy using a multi-mechanism strategy with multiple chemotherapy agents, it seems prudent to approach the management of HR/SR aGVHD with multiple efficacious agents, each targeting a different pathway, to best manage symptoms while preserving the graft-versus-tumor effect to the best of our ability.

## Conclusions

Although results are tempered by the administration of concomitant immunosuppressive agents and small patient numbers, MSCTC-0010 appears to be a safe and potentially efficacious agent for HR/SR aGVHD, with improved safety and an efficacy profile similar to FDA-approved ruxolitinib. This patient population has significant morbidity and mortality, which are as yet unmitigated by other agents; therefore, further clinical trials exploring the efficacy of MSCTC-0010 in larger populations and continued exploration of other novel agents are warranted.

## Supplementary Material

1

2

3

Supplementary material associated with this article can be found in the online version at doi:10.1016/j.jcyt.2025.102012.

## Figures and Tables

**Table 1:** Baseline patient characteristics.

Variable	Value (N = 24)
Sex (No. [%])	
Male	16 (67)
Female	8 (33)
Race/ethnicity (No. [%])	
White	21 (88)
Black	3 (12)
Age (median [range] [years])	59 (35–76)
Minnesota risk score (No. [%])	
High risk	1 (4)
Steroid-refractory	13 (54)
Both	10 (42)
Primary disease (No. [%])	
Acute myeloid leukemia	9 (38)
Myelodysplastic syndrome	3 (13)
Primary myelofibrosis	3 (13)
Secondary myelofibrosis	3 (13)
Chronic myelomonocytic leukemia	2 (8)
Chronic myeloid leukemia	2 (8)
Cutaneous T-cell lymphoma	1 (4)
Hodgkin lymphoma	1 (4)
GVHD therapy prior to WJMSCs (No. [%])	
Ruxolitinib	12 (50)
Extracorporeal photopheresis	3 (13)
Baseline GVHD organ involvement (No. [%])	
Skin	12 (50)
Upper GI tract	7 (29)
Lower GI tract	15 (63)
Liver	4 (17)
>1 organ	10 (42)

GI, gastrointestinal.

## Data Availability

Data are available from the corresponding author upon email request.
