# Supplementary figures and images for: A phase 1b study on the safety of Wharton’s jelly mesenchymal stromal cells in the treatment of acute graft-versus-host disease
Source: Cytotherapy. Author manuscript; Available in PMC 2026 Jul 13. (PMC13359252; doi:10.1016/j.jcyt.2025.102012)

**
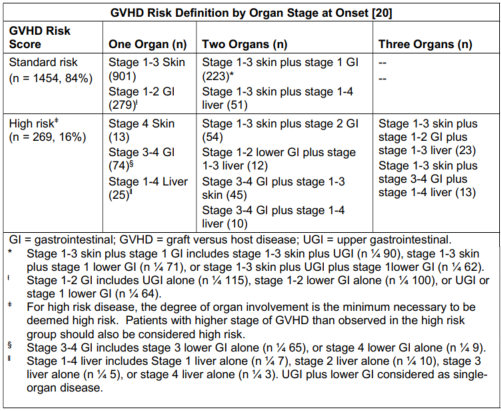
**

Supplement: 1 [file NIHMS2188476-supplement-1.docx]

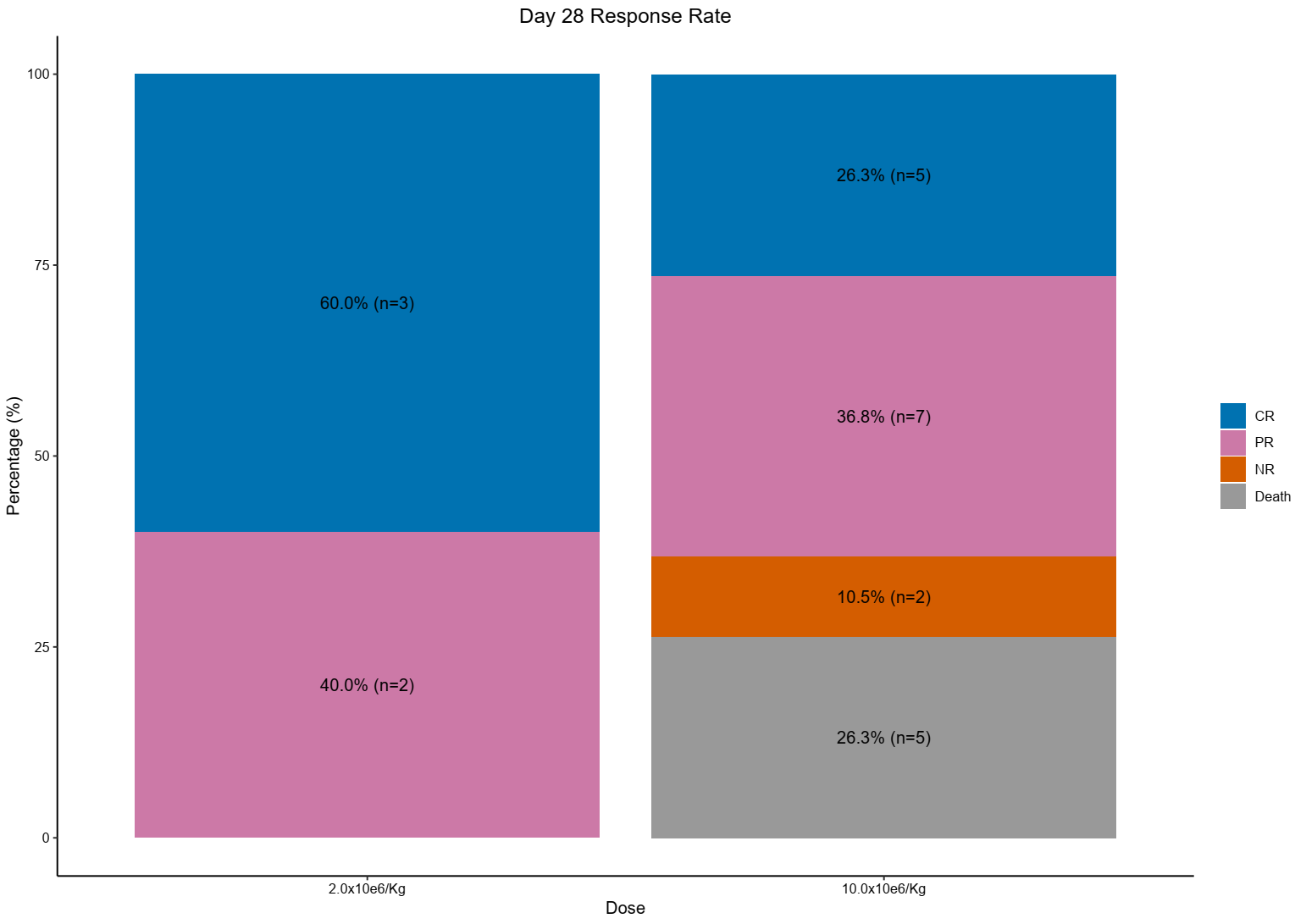

Supplement: 2 [file NIHMS2188476-supplement-2.docx]

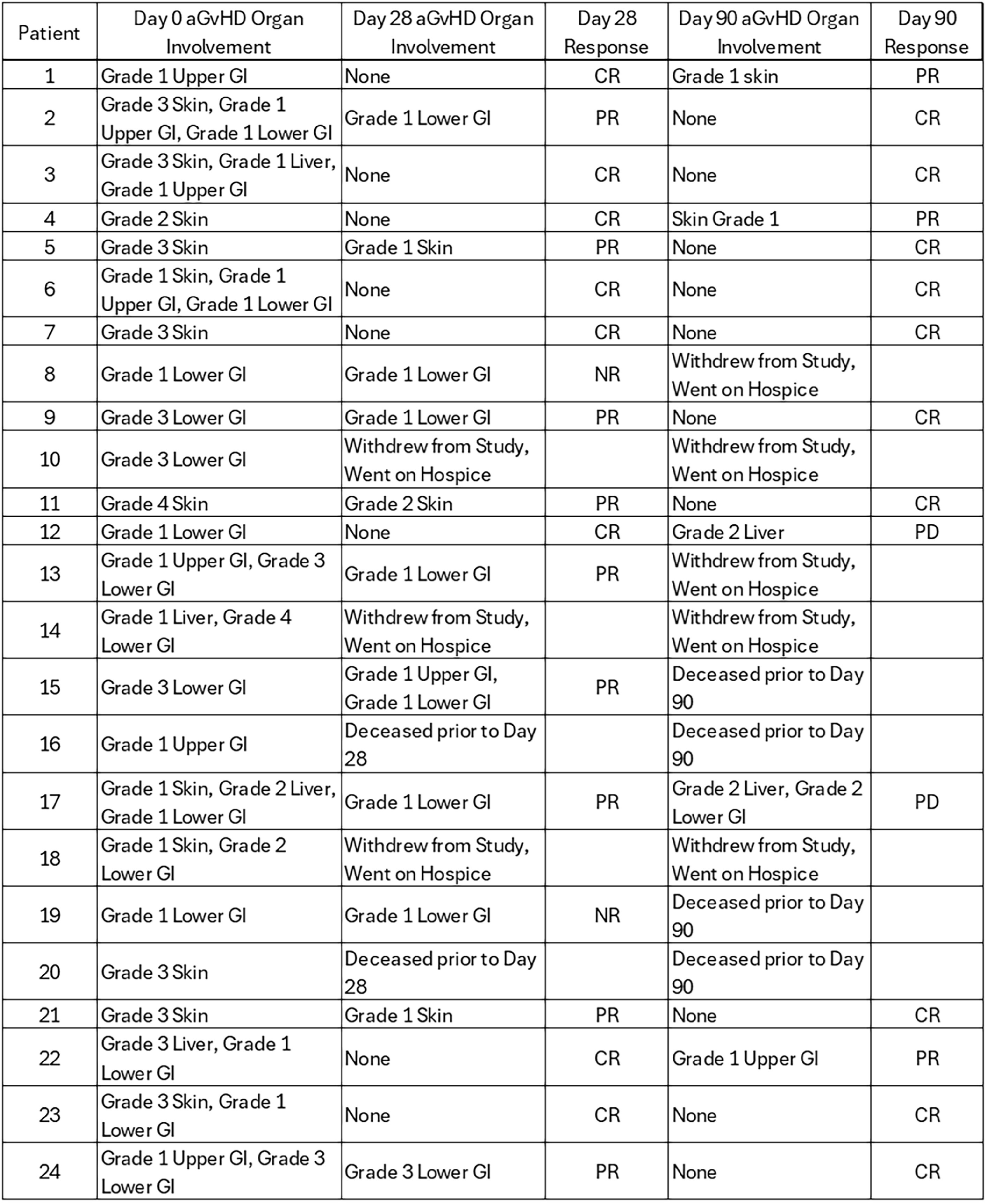

Supplement: 3 [file NIHMS2188476-supplement-3.jpg]
